# Targeting Neural Synchrony Deficits is Sufficient to Improve Cognition in a Schizophrenia-Related Neurodevelopmental Model

**DOI:** 10.3389/fpsyt.2014.00015

**Published:** 2014-02-14

**Authors:** Heekyung Lee, Dino Dvorak, André A. Fenton

**Affiliations:** ^1^Graduate Program in Neural and Behavioral Science, Downstate Medical Center, State University of NewYork, Brooklyn, NY, USA; ^2^Graduate Program in Biomedical Engineering, Downstate Medical Center, State University of New York and New York University Polytechnic School of Engineering, Brooklyn, NY, USA; ^3^The Robert F. Furchgott Center for Neural and Behavioral Science, Downstate Medical Center, State University of New York, Brooklyn, NY, USA; ^4^Center for Neural Science, New York University, New York, NY, USA

**Keywords:** neural synchrony, oscillations, schizophrenia, neurodevelopmental models, hippocampus, local field potentials, cognitive control, spike-wave discharge

## Abstract

Cognitive symptoms are core features of mental disorders but procognitive treatments are limited. We have proposed a “discoordination” hypothesis that cognitive impairment results from aberrant coordination of neural activity. We reported that neonatal ventral hippocampus lesion (NVHL) rats, an established neurodevelopmental model of schizophrenia, have abnormal neural synchrony and cognitive deficits in the active place avoidance task. During stillness, we observed that cortical local field potentials sometimes resembled epileptiform spike-wave discharges with higher prevalence in NVHL rats, indicating abnormal neural synchrony due perhaps to imbalanced excitation–inhibition coupling. Here, within the context of the hypothesis, we investigated whether attenuating abnormal neural synchrony will improve cognition in NVHL rats. We report that: (1) inter-hippocampal synchrony in the theta and beta bands is correlated with active place avoidance performance; (2) the anticonvulsant ethosuximide attenuated the abnormal spike-wave activity, improved cognitive control, and reduced hyperlocomotion; (3) ethosuximide not only normalized the task-associated theta and beta synchrony between the two hippocampi but also increased synchrony between the medial prefrontal cortex and hippocampus above control levels; (4) the antipsychotic olanzapine was less effective at improving cognitive control and normalizing place avoidance-related inter-hippocampal neural synchrony, although it reduced hyperactivity; and (5) olanzapine caused an abnormal pattern of frequency-independent increases in neural synchrony, in both NVHL and control rats. These data suggest that normalizing aberrant neural synchrony can be beneficial and that drugs targeting the pathophysiology of abnormally coordinated neural activities may be a promising theoretical framework and strategy for developing treatments that improve cognition in neurodevelopmental disorders such as schizophrenia.

## Introduction

Cognitive impairment is a core feature of schizophrenia but treatments to improve cognition are limited, in part because the physiological mechanisms of cognitive dysfunction are unclear. Recent interest has been directed toward understanding the abnormal synchrony of neural oscillations as a pathophysiological mechanism that underlies the cognitive impairments in schizophrenia (1–4). Dysfunctional neural synchrony in a wide range of frequencies, including the delta (5), theta (6), beta (7), and gamma ranges (8, 9), have been characterized in patients with schizophrenia. In animal models, disruption of long-range hippocampal–prefrontal synchrony has been reported while performing a working memory task in a genetic mouse model of schizophrenia (10) and during an open-field task in the maternal immune activation (MIA) neurodevelopmental model of schizophrenia (11). Abnormal hippocampal–prefrontal synchrony was also observed in the neonatal ventral hippocampus lesion (NVHL) neurodevelopmental model but this synchrony abnormality was not itself associated with cognitive impairment (12). In fact, aberrant neural synchrony may be a common pathophysiology responsible for impaired cognition in a variety of disorders (2, 13). Indeed, treatments that normalize neural network dysfunction without addressing the underlying cellular pathology have been sufficient to improve cognitive function in animal models of neurodegenerative diseases (14, 15). Such studies demonstrate it may be a feasible strategy to improve cognitive functional outcomes by normalizing aberrant neural network synchrony in cases where the network dysfunction is part of the causal chain from the disease etiology to the manifest cognitive symptoms.

We previously reported that NVHL rats, an established neurodevelopmental animal model of schizophrenia (16), have a cognitive control impairment that was associated with abnormal synchrony between the two hippocampi (12). We also detected hippocampal–prefrontal synchrony abnormalities in these rats during home-cage resting behaviors, but these abnormalities were absent during place avoidance training, and so not related to performance on the cognitive test. Clinically, the presence of hippocampal dysfunction is apparent in schizophrenia. Postmortem and neuroimaging studies indicate that the hippocampus is one of the brain regions most consistently altered in schizophrenia (17–22). Furthermore, functional magnetic resonance imaging studies show dysfunctional hippocampal activities are associated with poor cognitive performance in schizophrenia patients (23–25). Perhaps similar to the findings in NVHL rats, reduced inter-hippocampal functional coupling has also been observed between the left- and right-hippocampus areas in healthy carriers of the CACNA1C risk variant in bipolar disorder (26), but to our knowledge these measurements have not been carried out in schizophrenia subjects. Because neuroimaging studies do not have sufficient temporal resolution to detect functional synchrony at the 100-ms or faster timescales of the electrophysiological synchrony that underlies information processing between neural networks, it is unclear whether or not the inter-hippocampal abnormalities in NVHL rats mimics abnormalities in schizophrenia. Regardless, it is our opinion that animal models used to study schizophrenia do not sensibly mimic disease features, instead they are better thought of as being models for testing hypotheses that are relevant to understanding schizophrenia. Thus, we hypothesized that the abnormal hippocampal synchrony plays a role in the pathophysiology of cognitive control impairment in a schizophrenia-related neurodevelopmental animal model. As outlined below, in the present study, we tested the idea that attenuating this pathophysiology can improve cognition.

We, like others, have proposed the discoordination hypothesis, which asserts that cognitive impairment arises because of aberrant coordination of what may otherwise be normal neural activities (12, 27). The discoordination hypothesis is rooted in the idea that disrupted physiological coordination and synchronization of unitary neural processes impair the ability to selectively and dynamically activate and suppress information for organizing knowledge and perception into useable representations (1, 2, 27, 28). We use the term (dis)coordination to refer to the collective temporal organization of unitary neural activity such as action and synaptic and local field potentials (LFPs) at individual sites. Thus, (dis)coordination can be measured at different temporal and spatial scales. In the present work, we examined inter-hippocampal synchrony of LFP oscillations. These oscillations are an example of a long-range spatial scale neural interaction that involves an approximate order of 10^5^ neurons. These oscillations result from the multiple timescales of excitation–inhibition neural activity cycles that govern spiking within these networks (29). The discoordination hypothesis predicts that in the NVHL model, restoring the abnormal neural synchrony to normal will attenuate the cognitive control deficit. Accordingly, we took advantage of the hippocampal synchrony abnormality in NVHL rats, specifically the inter-hippocampal synchrony that was associated with cognitive performance, to evaluate the prediction. We examined whether normalizing the abnormal synchrony can attenuate the cognitive control deficit in the NVHL rats. We observed that hippocampal and neocortical LFPs in the NVHL rats sometimes resembled spike-wave discharges (SWDs), a sign of network hyperexcitability. We hypothesized that these aberrant neural activities arise from an excitation–inhibition imbalance that may underlie abnormal neural synchrony in the NVHL rats. Since SWD in epilepsy patients is treated with ethosuximide (2-ethyl-2-methylsuccinimide), an anticonvulsant that acts by blocking T-type Ca^2+^ channels, we studied whether ethosuximide would attenuate the abnormal SWD-like synchrony as well as impaired cognitive behavior and the associated neural synchrony that we have previously described in the NVHL model (12). We stress that our goal is to test the prediction of the discoordination hypothesis, not to suggest the use of ethosuximide as an antipsychotic. For comparison, we also examined the effects of the atypical antipsychotic olanzapine on neural synchrony and cognitive abnormalities in NVHL rats. Given that antipsychotics are not known to improve cognitive symptoms, the hypothesis predicts that olanzapine will not correct either the neural synchrony or cognitive abnormalities in NVHL rats.

We report that in NVHL rats, ethosuximide treatment attenuated the abnormal spike-wave activity, and most importantly, ethosuximide normalized the cognition-associated synchrony of theta and beta oscillations between the two hippocampi, and improved cognitive control in the place avoidance task. Olanzapine was less effective at reducing the abnormal spike-wave activity and normalizing the synchrony of oscillations between the two hippocampi and it did not improve cognitive control in NVHL rats, although it attenuated hyperlocomotion during the cognitive testing. These data are consistent with the discoordination hypothesis, and support the idea that drugs targeting the abnormal neural synchrony may be sufficient to rescue the cognitive deficits in schizophrenia-related neurodevelopmental animal models.

## Materials and Methods

All procedures were in compliance with National Institutes of Health guidelines and were approved by Downstate Medical Center’s Institutional Animal Care and Use Committee.

### Neonatal ventral hippocampal lesion

The procedure followed the manual provided by Lipska and Weinberger. We used the exact protocol that is published in our prior work (12). Briefly, timed-pregnant (13 or 14 days in gestation) female Long-Evans rats were obtained from Charles River Laboratories (Wilmington, MA, USA). Pups were born at the Downstate animal facility. On postnatal day 7 (P7), male pups were anesthetized by hypothermia. Bilateral puncture holes (relative to bregma AP: −3.0 mm, ML: ±3.5 mm) were made in the skull with a 30-Ga injection needle. A bilateral infusion (0.3 μl/side) of ibotenic acid solution (10 μg/μl) was delivered to each ventral hippocampus (relative to the skull surface DV: −5.0 mm). Control animals were treated identically except saline was injected instead of ibotenic acid. A subset of the NVHL and control rats were given drug treatments as described below. The behavioral and neural synchrony measurements from these rats were compared to untreated NVHL and control rats, the data from which were reported previously (12), although all data collection was concurrent amongst the different treatment groups.

### Drugs

Ibotenic acid, purchased from Sigma (St. Louis, MO, USA), was dissolved in artificial cerebrospinal fluid (ACSF) to a final concentration of 10 μg/μl, pH 7.6. Ethosuximide, purchased from Sigma (St. Louis, MO, USA), was dissolved in sterile 0.9% NaCl and a concentration of 100 mg/kg was injected i.p. Olanzapine, purchased from Toronto Research Chemicals Inc. (Toronto, ON, Canada), was dissolved in dimethyl sulfoxide (DMSO) and 1 mg/kg was injected i.p. Both ethosuximide and olanzapine were administered just prior to the start of behavioral training (see below) and based on published work, they were expected to have been bioactive for several hours, beyond the duration of behavioral training.

### Two-frame avoidance task

Five adult rats (P65) per group were trained on the two-frame task. The rat was placed on a circular 82 cm diameter, stainless steel rotating arena (Bio-Signal Group Corp., Brooklyn, NY, USA). The rat’s position was tracked using an overhead camera and software (Tracker, Bio-Signal Group Corp., Brooklyn, NY, USA). All animals were habituated to the rotating arena the day before the start of active place avoidance training during two 10-min sessions without any shocks. On the following training trials, whenever the rat on the rotating arena entered a 60° shock zone that was defined in room coordinates, a mild constant current (60 Hz, <0.4 mA, 500 ms) foot shock was delivered. Foot shock was repeated every 1.5 s until the rat left the shock zone. Each place avoidance trial was 10 min and the interval between the trials was at least 10 min. A total of eight training trials were given on the training day. The drugs were injected i.p. just prior to the first avoidance training trial. Avoidance was measured by counting the number of times the rat entered the shock zone.

### Local field potential and electroencephalogram recordings

All rats were housed individually, exposed to a 12-h light/dark circadian cycle, and had *ad libitum* access to water and food. When rats were P55-57, electrodes were surgically implanted bilaterally. Rats were given post-operative Ketofen (5 mg/kg) for 2 days after surgery and allowed to recover from the surgical procedure for 7 days. LFPs in the dorsal hippocampus (AP: −4 mm; ML: ±2.5 mm; DV: 3 mm) and in the medial prefrontal cortex (mPFC; AP: +3 mm; ML: ±1 mm; DV: 4 mm) were recorded by implanting 75-μm Nichrome wires that were attached to a Millmax connector. The epidural cortical electroencephalogram (EEG) was recorded with a screw electrode in the frontal and parietal bones of each hemisphere. All electrodes were referred to an electrode implanted in the cerebellar white matter (AP: −10 mm; ML: +2 mm; DV: 3 mm), which seemed to be normal in NVHL rats. Recordings were made with a wireless digital telemetry system (Bio-Signal Group, Brooklyn, NY, USA). Recordings were made while the rats were performing the two-frame avoidance task and in the home-cage (for 2–3 h) during the light cycle.

The signals at the electrode connector were amplified (300 times), low-pass filtered (6 kHz) and digitized (24-bits, 12 kHz using delta-sigma analog-digital convertors). The digital signals were transmitted wirelessly to a recording system (dacqUSB, Axona Ltd., St. Albans, UK) for band-pass filtering (1–500 Hz), digital amplification and down-sampling (16-bits, 2000 Hz) using digital signal processors. The digital electrophysiological data were stored on computer hard drives for off-line analysis. The channels were marked if they had saturated signals and were excluded from further analysis if the proportion was substantial. At the end of training and recording, the rats were trans-cardially perfused and the brains removed for histological verification of the electrode locations.

### Power spectra

Power spectra measure the amplitude of frequency-specific oscillations that arise when spatially patterned synaptic potentials are coincident in time. These oscillations are locally generated phenomena. As such, an oscillation itself may not be a strong indication of neural synchrony as it relates to the concept of neural coordination (1, 27). Instead, power spectra are an estimate of the amount and magnitude of patterns of oscillation-generating synaptic potentials that are the fundamental components of neural coordinating processes. The patterns of synaptic potentials must themselves be temporally and spatially coordinated within and between neural networks to mediate the neural computations and information processing upon which cognition is based (29).

**Figure 1:**
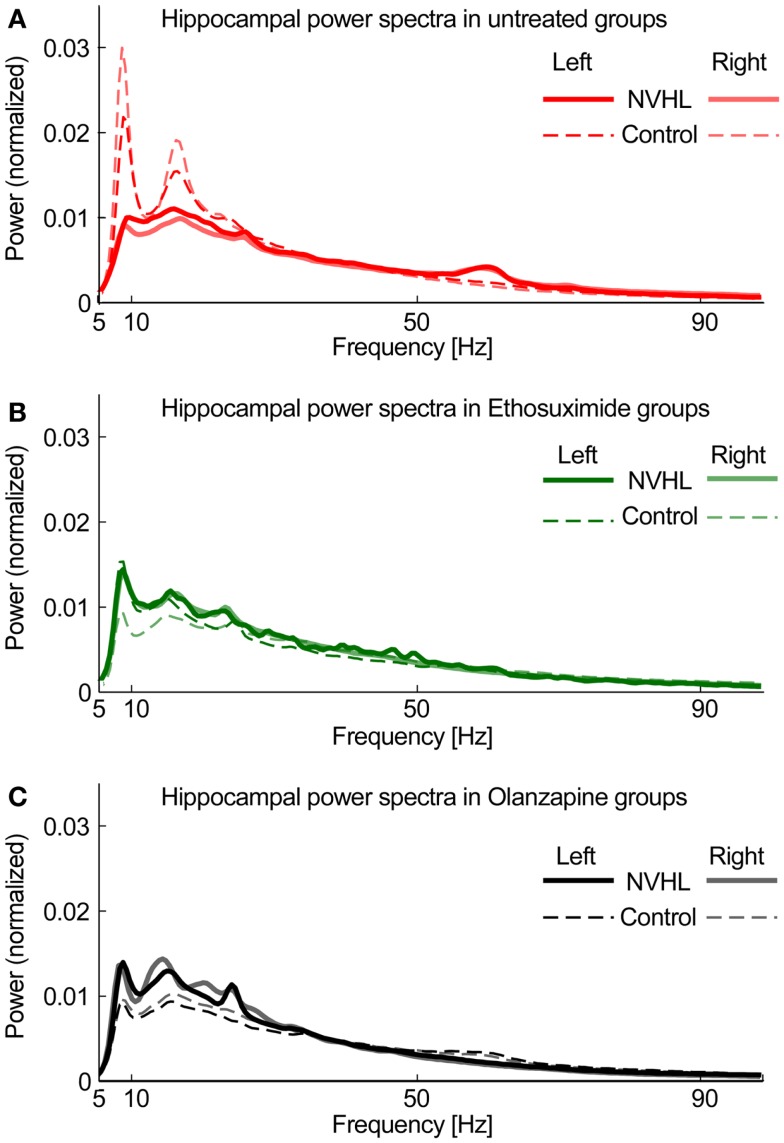
**Hippocampal power spectra**. **(A)** Power spectra density plot for untreated groups averaged across all subjects for NVHL (thick line) and control (thin line) computed independently for left (opaque line) and right (semi-transparent line) hippocampi. **(B)** Power spectral density plot for Ethosuximide-treated animals. **(C)** Power spectra density plot for Olanzapine-treated animals. The similarities between the corresponding left and right spectra is a common observation, is independent of phase synchrony, and is only indirectly related to neural coordination in that neural oscillations are the fundamental, unitary components of neural coordination processes such as phase synchrony and cross-frequency coupling (30).

To compute power spectra, LFP signals were first normalized by dividing the signal by its RMS value. This step minimized electrode-specific signal properties caused by variability in electrode location or impedance. The signal was processed using 5 s long non-overlapping windows. For each window power spectral density (PSD) was computed using the “pwelch” Matlab function. The spectral peak at 60 Hz was removed and the PSD was interpolated between 58 and 62 Hz. The resulting PSD was normalized by dividing it by its sum so the total power was equal to 1. Based on the spectral profile, we then defined bands of interest for frequency-specific phase-locking analysis. We excluded the delta band (0–3 Hz) because of its attenuation by the hardware DC-removal filter (Figure 1). We defined the theta band as 5–15 Hz, beta as 20–30 Hz in order to exclude the strong second theta harmonic (Figure 1), slow gamma 30–55 Hz and fast gamma as 65–100 Hz (excluding the 60-Hz region). Figure 1 shows that the left and right hippocampal spectra are similar, as expected, and that the spectra are attenuated in NVHL rats. Both ethosuximide and olanzapine only caused a marginal increase in the broadband spectral power. This confirmation of stable spectral power provides the starting point for the present study, which focuses on measuring how these component oscillations are coordinated between brain regions.

### Phase-locking value

The coordination of neural signals at a pair of electrodes was estimated by computing the phase-locking value (PLV). Eight LFP channel pairs were selected for PLV analysis. They were the left- and right-hippocampi; left- and right-mPFC; left-hippocampus and left-mPFC; right-hippocampus and right-mPFC, as well as the inter-hemispheric and associational epidural electrode pairs. The PLV at a pair of electrodes was computed using custom software written in Matlab, based on a published algorithm (31). The phase of a signal at time *t* and sample *n* ϕ(*t, n*) was obtained by filtering the signal with a narrow-band finite input response (FIR) filter using a zero phase-shift filtering algorithm followed by the Hilbert Transform. The filters were designed using the Matlab filter design toolbox. Filters in the range 0–100 Hz had fixed bandwidth of 5 Hz. For bands of interest extending over several 5 Hz bands the PLV across all such bands were averaged. Given a pair of LFP signals of *N* samples, the PLV was defined as follows:

$$\text{PLV}=\frac{1}{N}\left|\sum_{n=0}^{N}\exp\;[i\theta (t,n)]\right|,$$

where θ(*t, n*) is the phase difference ϕ_1_(*t, n*) − ϕ_2_(*t, n*) between the two signals at time *t* sample *n*; *N* is the total number of samples; and *i* is the imaginary unit. Prior work showed that the intracranial electrode-pair specific PLVs at the four (theta, beta, slow gamma, fast gamma) frequency bands were different between NVHL and control rats (12). We made these comparisons between the NVHL and control treatment groups to determine if the drug treatment normalized neural synchrony between the particular electrode pairs. In addition, the PLVs for the full set of electrode pairs were studied to look for neural synchrony patterns that distinguished the NVHL and control and the drug-treatment groups. The set of electrode-pair and frequency-specific PLVs for each recording session was treated as a vector with dimension 32 (8 electrode pairs × 4 bands). Principal component analysis (PCA) was performed to reduce the dimensionality and identify the most useful measures of synchrony. Each session vector of PLVs (eight sessions per rat) was plot in the coordinate system of the first three principal components to visualize the group patterns. The first six principal components explained over 90% of the variance and so for quantification we only considered the first six principal components. We quantified the distance between the vectors in each session group by computing the 6-D Euclidean distance between all pairs of data points within a treatment group, and also between all pairs of data points against a reference treatment group. The untreated control and untrained NVHL groups as well as the ethosuximide-treated NVHL and the olanzapine-NVHL groups each served as reference groups for the comparisons. These pair-wise group distances were compared by two-sample Student’s *t*-tests that were adjusted for multiple comparisons using the Bonferroni method.

### Automatic detection of spike-wave activity

We used an algorithm to identify activity that resembled epileptiform SWDs, but are observed in normal Long-Evans rats without seizures (32). Each channel was first examined for signal saturation and only data segments with brief (<50 ms) saturations were considered for further analysis. Brief saturations in the LFP were allowed so spike-wave complexes with a large spike component were not removed from the analysis. We manually marked epochs of spike-wave events using the EEGLAB toolbox for Matlab. These epochs were used to calculate and optimize the detection parameters for the automatic classifier of spike-wave events. The classifier is a modified version of a published algorithm (33).

**Figure 2:**
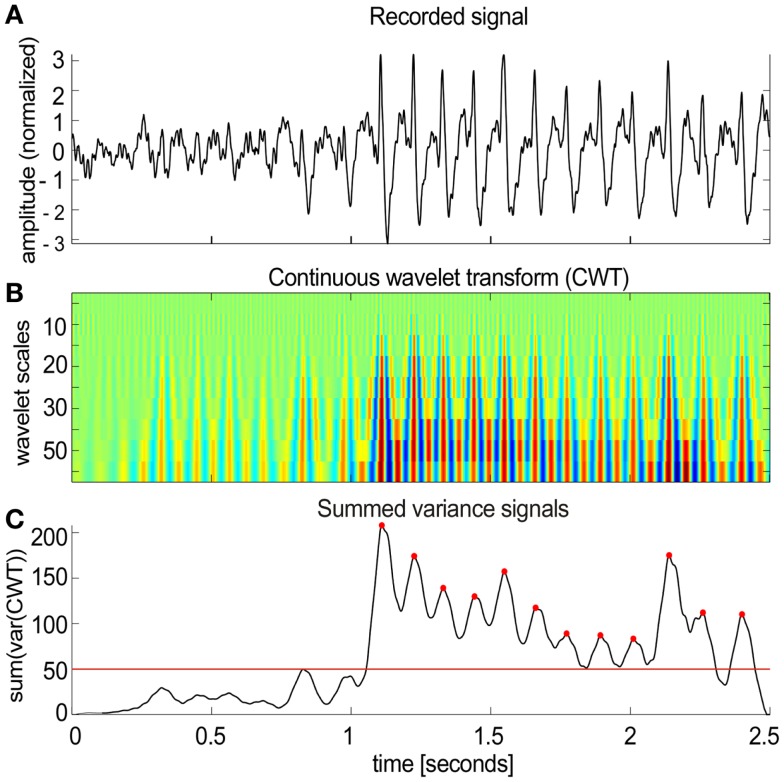
**Spike-wave activities in NVHL and control rats**. **(A)** A segment of the cortical EEG with spike-wave activity. An automatic classifier was used to detect these events. **(B)** The first step in the classifier was to transform the recorded signal using the continuous wavelet transform (CWT). **(C)** Then a sliding variance was computed for each wavelet scale. All the variances were summed across all scales resulting in a single variance profile over time (50 ms smoothing was applied to the resulting signal). SWD candidates were detected as threshold crossings [red line in **(C)**]. Each marked segment was tested for periodic spiking activity by detecting local maxima [red dots in **(C)**]. Only the detected segments that contained at least 50% of inter-spike intervals in the range of 2–10 Hz were included in further analysis.

The algorithm worked in several steps (Figure 2):

(1) Local field potential signals (Figure 2A) were transformed (Figure 2B) using the continuous wavelet transform (CWT) from the Wavelet Toolbox in Matlab (Morlet wavelets, scales 5–50 with step of 5 corresponding to pseudo-frequencies 16–160 Hz).
(2) The sliding variance (50 ms long window) was computed for each scale and all variances were summed across all scales resulting in a single variance profile over time.
(3) Candidate spike-wave events were marked as threshold crossings (red line in Figure 2C) of the summed variance series.
(4) Gaps between individual marked segments shorter than 0.75 s were filled to create longer segments.
(5) After filling the gaps, all marked segments that were shorter than 1 s were removed from further analysis.
(6) Each marked segment was tested for periodic spiking activity by detecting local maxima (red dots in Figure 2C) in the summed variance signal with 50 ms smoothing applied. The inter-spike intervals for each pair of sequential maxima were computed. Only those marked segments that contained at least 50% of inter-spike intervals in the range of 2–10 Hz (the typical range of SWDs) were considered as spike-wave segments.
(7) The process was repeated several times with different parameters and the classified event segments were compared with manually marked events to tune the classifier.

### Analysis of spike-wave event features

Only manually marked segments were included in this analysis to avoid distortion by borderline spike-wave-like events that were detected by the automatic algorithm but did not necessarily share all the features such as clear spike and wave components. The subjectively selected events were identified on the basis of the classic spike-wave morphology, by an observer blind to the origin of the EEG. Thus the manual selection of spike-wave events resulted in a dataset of events with a bias to less specificity and more selectivity.

The manually marked events were first low-pass filtered with a cut-off frequency of 100 Hz. The filter removed the high frequency components that could cause errors in feature extraction. Local maxima were then detected in the signal and for each maximum, its preceding and following local minima were found. The spike components of the events were then defined as local maxima, surrounded by local minima with a maxima–minima amplitude of at least 400 μV and with a spike width (defined below) of at least 50 ms. Spike sequences (for computing the inter-spike time) were defined as continuous spikes with a frequency between 5 and 13 Hz. The feature analysis was repeated several times to optimize the parameters for the component detection.

**Figure 3:**
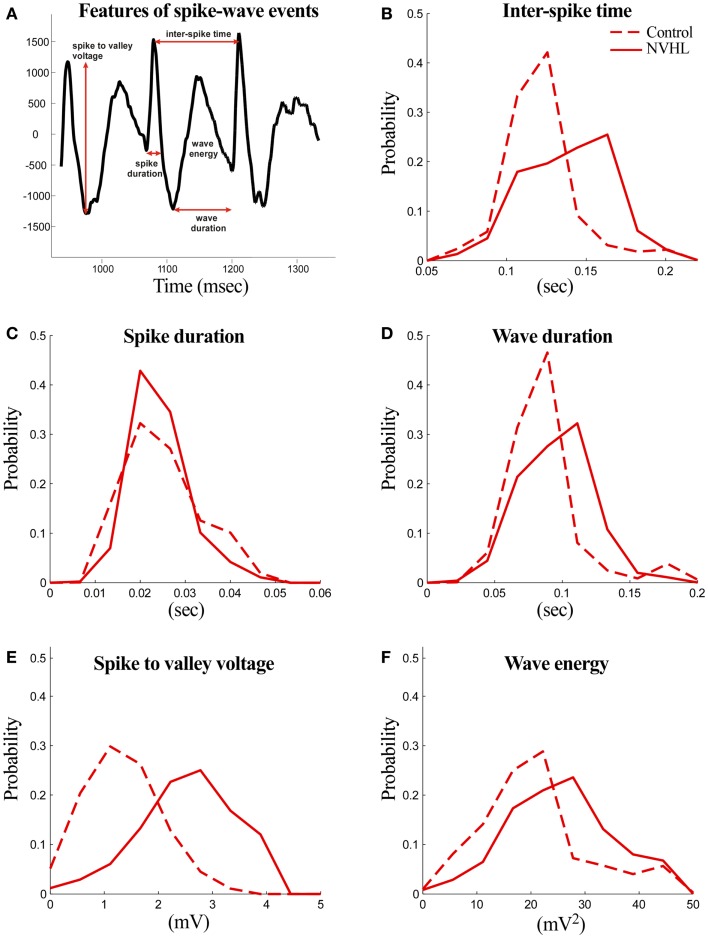
**Abnormal features of the spike-wave activities in NVHL rats**. **(A)** Three spike-wave cycles are shown to illustrate the features that were measured. The analyzed features include spike duration, inter-spike time, wave duration, spike-to-valley voltage, and inter-spike time. Comparison of the spike-wave complex features in NVHL and control rats: **(B)** inter-spike time, **(C)** spike duration, **(D)** wave duration, **(E)** spike-to-valley voltage, and **(F)** wave energy. Distributions in each feature looked distinct, except in spike duration, and this was confirmed statistically by the two-sample Kolmogorov–Smirnov test in which the null hypothesis that the control and NVHL distributions were drawn from the same underlying distribution was rejected (*p* < 0.05).

The following features were computed for both NVHL and control rats (Figure 3):

(1) Spike duration: the duration of the spike measured at the level of either the preceding or the following local minimum with the higher voltage.
(2) Spike-to-valley voltage: the voltage between the spike peak and the following local minimum (valley).
(3) Inter-spike time: the time between two successive spikes.
(4) Wave duration: the duration of the wave component measured from the local minimum (valley) following the spike component and the local minimum preceding the next spike component.
(5) Wave energy: the energy of the wave component computed as the sum of squares of amplitudes.
(6) Spike-wave event prevalence: only the automatically classified segments were included in this analysis. Prevalence was estimated as the total time spike-wave events were detected and divided by the total amount of time that was analyzed.

### Statistical analysis

Group differences were compared by ANOVA or Student’s *t*-test. The PLV between groups and across frequency bands were compared by two-way ANOVA. Repeated-measures were not used because the data were independently and differentially filtered for computing the synchrony estimates at each frequency band. Significance was set at *p* < 0.05, and Tukey’s HSD *post hoc* tests were used as appropriate. The two-sample Kolmogorov–Smirnov test was used to compare the probability distributions of spike-wave event features in the NVHL and control groups. The distributions were generated by selecting a constant subsample of spike-wave events from each animal such that each subject contributed equally to the distribution. The resulting distribution was then divided by its sum to generate probability distributions that are normalized against different numbers of subjects in the NVHL and control groups.

## Results

### Abnormal spike-wave activities in the EEG of NVHL rats

Before focusing on the main aim of this study, to investigate cognition-related inter-hippocampal phase synchrony during active place avoidance cognitive behavior, we began by monitoring the field potential recordings from NVHL and control rats as they behaved in their home-cage. In the home-cage, both groups of rats were still or moving <0.5 cm/s about 80% of the time based on estimates from concurrent video tracking. The most conspicuous feature was the occasional presence of a large amplitude series of spike-wave events. We were surprised to find these events and will call them spike-wave activity. They typically occurred in trains of ~6 Hz (Figure 2A). These events have been observed in the cortical EEG of normal Long-Evans rats during stillness and are a sign of heightened synchrony of cortical neural activity, perhaps the rat analog of the human alpha-like mu rhythm (32, 34). We automatically detected the individual events and confirmed by visual inspection that the algorithm properly detected the events (Figures 2B,C). Recordings from the epidural cortical screw electrodes were used for analysis because signals from the epidural cortical electrodes showed the strongest spike-wave waveforms across all animals. We analyzed a total of nine NVHL and seven control rats during home-cage behavior to estimate the prevalence of the spike-wave activity. Each rat was recorded for 3–5 h in the home-cage. The spike-wave activity was detected in six of nine NVHL rats and three of seven control rats. The prevalence was 3% across all the NVHL rats and 2% in all the control rats. In total, 1514 spike-wave events were detected in NVHL rats and 397 spike-wave events were detected in control rats, suggesting that these events were more common in NVHL rats.

We then measured various features of the spike-wave events in both NVHL and control rats (Figures 3A–F). The distributions of NVHL and control groups looked distinct in all the features except in spike duration. These were confirmed statistically by the two-sample Kolmogorov–Smirnov test because the null hypothesis that the control and NVHL distributions are drawn from the same underlying distribution was rejected (*p* < 0.05) for all features. These events tended to be larger amplitude and more frequent in NVHL rats in comparison to the control rats. Since we have previously characterized a cognition-related abnormality in neural synchrony in NVHL rats, in the framework of the discoordination hypothesis, these observations of abnormal synchrony suggested to us that reducing the spike-wave activities could improve cognition and related neural activity in NVHL rats.

### Ethosuximide reduced the prevalence of abnormal spike-wave activities

We then evaluated whether the spike-wave activities could be attenuated by ethosuximide, an anticonvulsant that is an antagonist of T-type calcium channels (35). It is used mainly to treat absence seizures. Given the framework that animal models are best utilized to test hypotheses about schizophrenia and that it is unwise to assume that models themselves mimic the disease (36), we reasoned that since ethosuximide is effective at suppressing epileptiform SWDs, then it may be also be effective on the spike-wave activities in the NVHL rats. Since it might suppress this specific form of abnormal neural synchrony by rebalancing excitation and inhibition, it occurred to us that ethosuximide could also act to reduce the cognition-related abnormal neural synchrony, which according to the discoordination hypothesis would attenuate the cognitive deficit observed in these rats.

For comparison, we also chose to study the effects of olanzapine, a compound with antagonist actions at dopaminergic, muscarinic, adrenergic, GABAergic, and histaminergic receptors. It is one of the most widely used second-generation antipsychotic drugs. These drugs were designed to suppress hyperlocomotion in animal models and are prescribed to reduce the positive symptoms of psychosis, but are not considered to be effective on cognitive symptoms.

**Figure 4:**
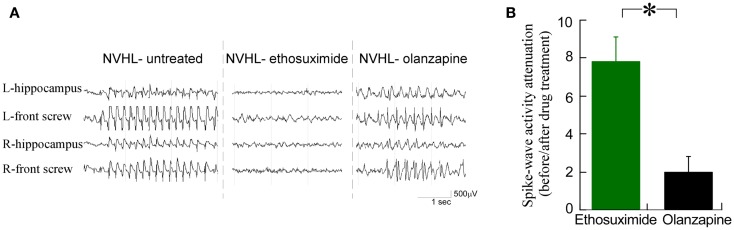
**Ethosuximide reduced the prevalence of abnormal spike-wave activities in NVHL rats**. **(A)** Raw EEG traces. Ethosuximide reduced the occurrence of spike-wave activity while olanzapine did not. **(B)** Quantification of the spike-wave activity prevalence before and after drug treatment. There was an eightfold reduction of the prevalence after ethosuximide treatment, while only a twofold attenuation of the prevalence after olanzapine treatment in NVHL rats (*t*_4_ = 3.79, *p* = 0.02).

We observed that ethosuximide reduced the spike-wave activity much more than olanzapine (Figure 4A). There was an eightfold reduction in the prevalence of spike-wave activity after ethosuximide (100 mg/kg i.p.) treatment, while only a twofold reduction in spike-wave activity in the NVHL rats (Figure 4B) after a dose of olanzapine (1 mg/kg i.p.) that is sufficient to attenuate sensorimotor deficits such as hyperlocomotion in the NVHL (see Figure 7C below) and acute PCP animal models (37). These observations motivated us to test whether ethosuximide can attenuate the abnormal cognition-related neural synchrony that we have previously characterized in NVHL rats (12).

### Ethosuximide attenuated abnormal inter-hippocampal synchrony

**Figure 5:**
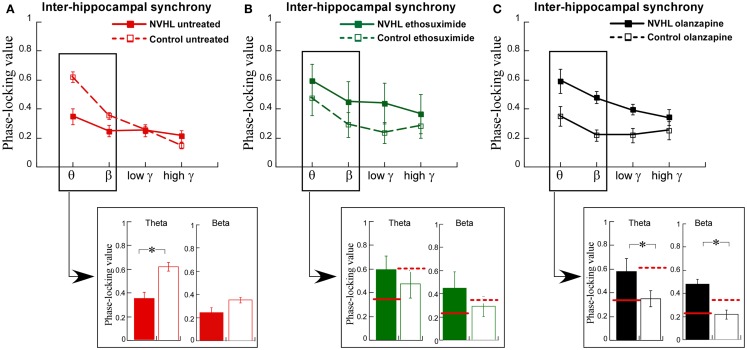
**Ethosuximide attenuated abnormal cognitive control-associated inter-hippocampal synchrony**. **(A)** Adult NVHL rats had lower inter-hippocampal synchrony compared to control rats (two-way ANOVA group: *F*_1,32_ = 8.87, *p* = 0.006; frequency: *F*_3,32_ = 23.6, *p* < 0.0001; interaction: *F*_3,32_ = 7.68, *p* = 0.0005). Inter-hippocampal theta (*t*_8_ = 3.98, *p* = 0.004) and beta (*t*_8_ = 2.30, *p* = 0.05) synchrony was weaker in the NVHL rats compared to the controls. **(B)** Ethosuximide treatment attenuated the inter-hippocampal synchrony difference between the NVHL and control rats (two-way ANOVA group: *F*_1,32_ = 3.05, *p* = 0.09; frequency: *F*_3,32_ = 1.42, *p* = 0.25; interaction: *F*_3,32_ = 0.10, *p* = 0.96). There was no theta (*t*_8_ = 0.73, *p* = 0.49) or beta (*t*_8_ = 0.94, *p* = 0.37) synchrony difference. **(C)** Olanzapine treatment increased synchrony in NVHL rats and decreased synchrony in control rats, resulting in an inter-hippocampal synchrony difference between the groups (two-way ANOVA group: *F*_1,28_ = 28.2, *p* < 0.0001; frequency: *F*_3,28_ = 6.20, *p* = 0.002; interaction: *F*_3,28_ = 1.53, *p* = 0.23). Inter-hippocampal theta (*t*_7_ = 2.94, *p* = 0.02) and beta (*t*_7_ = 4.43, *p* = 0.003) synchrony was higher in the NVHL rats compared to the controls.

We then turned to the main effort of this research. We recorded LFPs while the rats performed the two-frame active place avoidance task. We previously identified that oscillations in the theta and beta ranges was less synchronized between the left and right dorsal hippocampi in NVHL rats than in control rats and that this inter-hippocampal synchrony was correlated with effective place avoidance behavior (12). Inter-hippocampal theta and beta synchrony was weaker in the NVHL rats compared to the controls (Figure 5A), which was already shown in prior work (12). We administered ethosuximide and olanzapine just prior to the first place avoidance training trials in new NVHL and control groups. The difference in inter-hippocampal synchrony between the NVHL and control rats was attenuated by ethosuximide (Figure 5B), suggesting that ethosuximide normalized inter-hippocampal synchrony in NVHL rats. Following olanzapine, inter-hippocampal synchrony was different between the NVHL and control groups (Figure 5C). However, this difference appeared because the drug increased synchrony in the NVHL rats and decreased synchrony in the control group, especially in the theta and beta bands. Indeed, after olanzapine, synchrony in the NVHL rats was greater than in the treated control rats. Olanzapine also increased inter-hippocampal synchrony in the faster frequency gamma bands. This increase was in excess of the synchrony seen in untreated control rats, which in contrast to ethosuximide, is why olanzapine did not restore normal synchrony.

**Figure 6:**
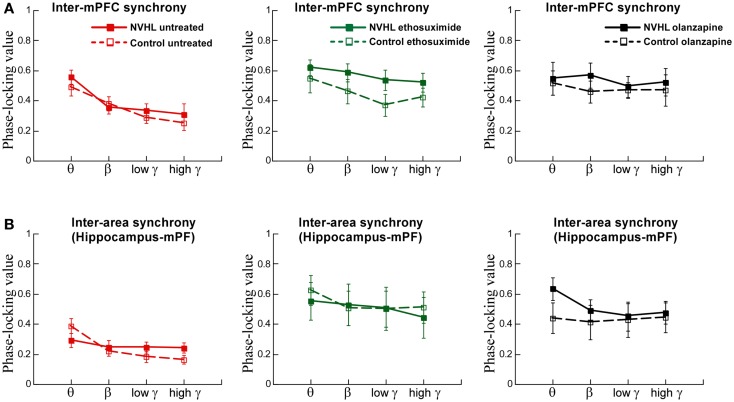
**Inter-mPFC or inter-hippocampus-mPFC phase synchrony was not different in the NVHL and control groups during active place avoidance**. **(A)** Inter-mPFC synchrony was frequency-dependent but not different between the NVHL and control rats in the untreated groups (two-way ANOVA group: *F*_1,32_ = 0.92, *p* = 0.34; frequency: *F*_3,32_ = 8.97, *p* = 0.0001; interaction: *F*_3,32_ = 0.33, *p* = 0.81). The inter-mPFC synchrony was greater in the NVHL rats in the ethosuximide-treated group and the frequency dependence was lost due to increased synchrony at the gamma frequencies (middle: two-way ANOVA group: *F*_1,32_ = 5.42, *p* = 0.03; frequency: *F*_3,32_ = 1.41, *p* = 0.26; interaction: *F*_3,32_ = 0.16, *p* = 0.92). In the olanzapine-treated rats, the group and frequency dependencies were no longer observed because the drug increased synchrony at all frequencies we assessed (right: two-way ANOVA group: *F*_1,28_ = 1.43, *p* = 0.24; frequency: *F*_3,28_ = 0.40, *p* = 0.76; interaction: *F*_3,28_ = 0.11, *p* = 0.95) groups. **(B)** Inter-area synchrony (between mPFC and hippocampus) was frequency-dependent but not different between the NVHL and control rats in the untreated group (left: two-way ANOVA group: *F*_1,32_ = 0.43, *p* = 0.51; frequency: *F*_3,32_ = 5.29, *p* = 0.005; interaction: *F*_3,32_ = 2.05, *p* = 0.13). There were no group or frequency dependencies in the ethosuximide-treated rats because the drug increased synchrony at all frequencies to the level of the maximal (theta) inter-hippocampal synchrony (middle: two-way ANOVA group: *F*_1,32_ = 0.11, *p* = 0.74; frequency: *F*_3,32_ = 0.30, *p* = 0.83; interaction: *F*_3,32_ = 0.07, *p* = 0.97). The olanzapine-treated NVHL rats had greater synchrony at all frequencies and there was no frequency dependence because the drug also increased synchrony at all frequencies (right: two-way ANOVA group: *F*_1,28_ = 1.22, *p* = 0.28; frequency: *F*_3,28_ = 0.26, *p* = 0.85; interaction: *F*_3,28_ = 0.19, *p* = 0.90) groups.

We also examined inter-hemispheric synchrony between electrodes in the mPFC (Figure 6A). The synchrony of LFP oscillations between the left and right-mPFC was greater for slower frequencies than faster frequencies but not different between the NVHL and control groups. After ethosuximide, the frequency dependence on the magnitude of synchrony between the left and right-mPFC was no longer observed. This was in large part because ethosuximide increased synchrony at the faster gamma frequencies, almost to the level of the slower beta frequencies. This effect was more pronounced in the NVHL animals. Olanzapine had a similar effect on synchrony between the left and right-mPFC sites such that there was no longer a detectable difference in the magnitude of synchrony in the different frequency bands and the NVHL and control groups were also indistinguishable.

Finally, we examined synchrony between the mPFC and dorsal hippocampus of one hemisphere (Figure 6B). Synchrony was generally less than it was between the two hippocampi or between the two mPFC. There was greater synchrony in slower frequency bands than the faster gamma bands. There was, however, no difference between NVHL and control animals. After ethosuximide, synchrony increased, especially in the faster bands, for both the NVHL and control animals. This removed the effect that synchrony was greater at slow frequencies. The NVHL and control groups were also indistinguishable after ethosuximide. Olanzapine increased synchrony in both NVHL and control rats but the increase was greater for the NVHL rats. Synchrony increased in all bands so there was no effect of the frequency band. Clearly, both ethosuximide and olanzapine altered the synchrony of oscillations in the LFPs of control and NVHL rats.

### Ethosuximide attenuated the cognitive control impairment of NVHL rats

**Figure 7:**
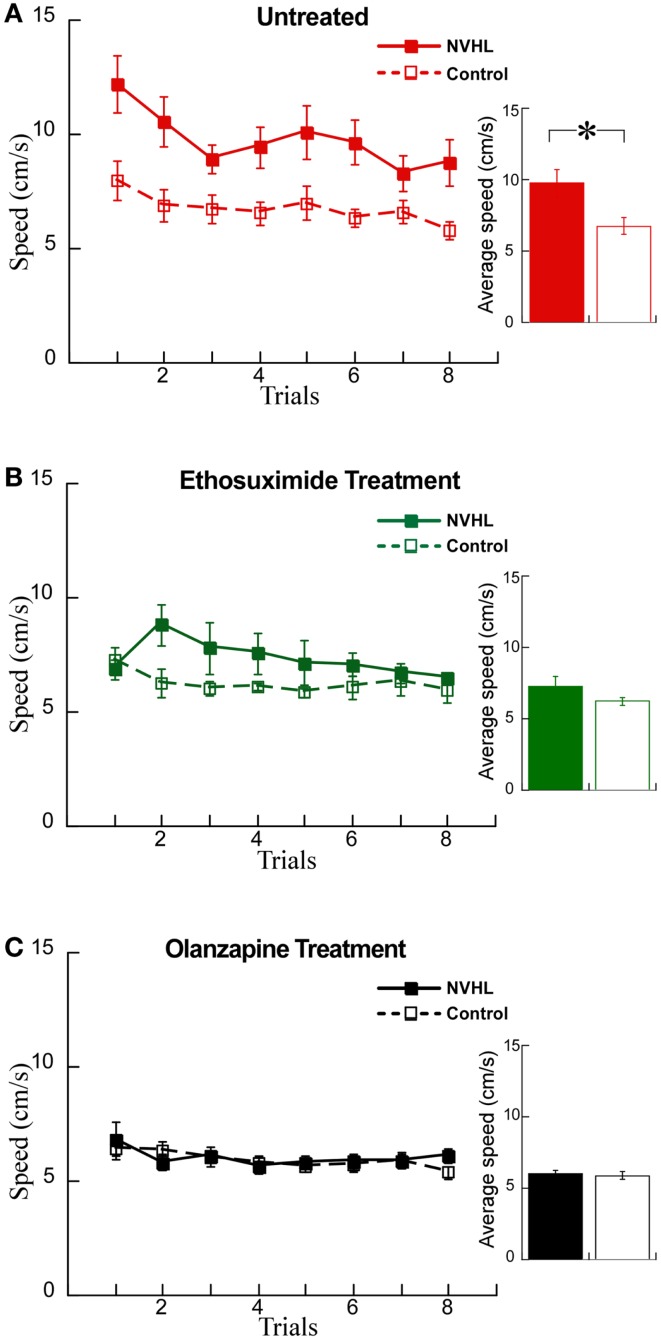
**Olanzapine and ethosuximide reduced hyperactivity in NVHL rats**. **(A)** In the untreated group, the NVHL rats were hyperactive compared to control rats while performing the two-frame avoidance task (performance curve: two-way repeated-measures ANOVA group: *F*_1,64_ = 53.34, *p* < 0.0001; trial: *F*_7,64_ = 2.37, *p* = 0.03; interaction: *F*_7,64_ = 0.46, *p* = 0.86); inset: average speed: *t*_8_ = 2.78, *p* = 0.02). **(B)** Ethosuximide treatment reduced hyperactivity in the NVHL rats compared to untreated NVHL rats, and showed a statistical difference when compared to control rats (performance curve: two-way repeated-measures ANOVA group: *F*_1,64_ = 11.68, *p* = 0.001; trial: *F*_7,64_ = 0.86, *p* = 0.54; interaction: *F*_7,64_ = 1.09, *p* = 0.38); inset: average speed: *t*_8_ = 1.5, *p* = 0.17). **(C)** Olanzapine treatment reduced hyperactivity so there was no longer a difference from control rats (performance curve: two-way repeated-measures ANOVA group: *F*_1,64_ = 0.36, *p* = 0.55; trial: *F*_7,64_ = 1.37, *p* = 0.23; interaction: *F*_7,64_ = 0.48, *p* = 0.85); inset: average speed: *t*_8_ = 0.30, *p* = 0.77).

We examined whether these changes in synchrony were associated with changes in behavior using the active place avoidance task variant that allows evaluation of the ability to use relevant information and ignore irrelevant information, what is known as cognitive control. We first examined the effects of the drugs on locomotor hyperactivity (Figure 7A), which is a feature of NVHL rats that we have previously observed during the place avoidance task (12). Indeed, hyperactivity has been widely used to model the positive symptoms of schizophrenia and the effect of olanzapine in reducing hyperactivity has been shown in a variety of schizophrenia-related animal models (38, 39). Ethosuximide significantly reduced hyperactivity in NVHL rats compared to control rats (Figure 7B). As expected, olanzapine also reduced hyperactivity in NVHL rats compared to controls rats, so that the two groups were indistinguishable (Figure 7C).

**Figure 8:**
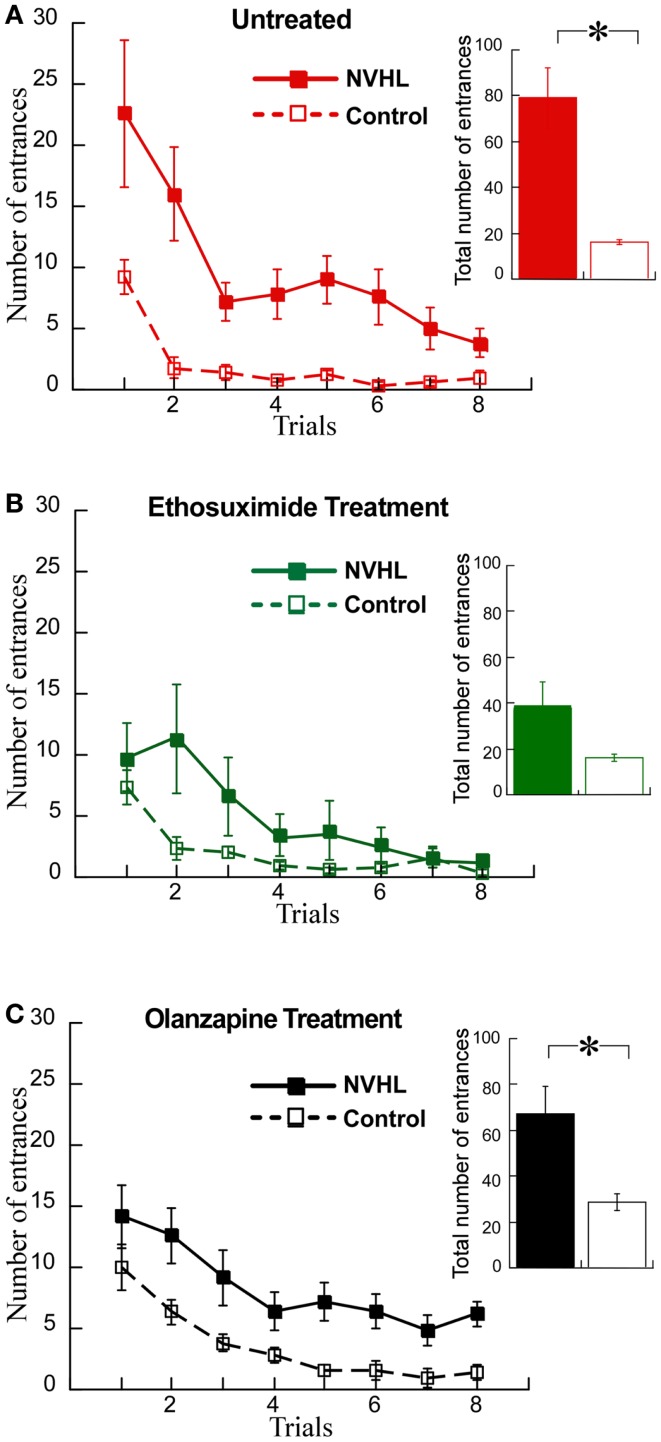
**Ethosuximide attenuated the cognitive control impairment of NVHL rats**. **(A)** In the untreated group, the NVHL rats were slower to learn the two-frame task and showed an impairment when compared to the control rats (learning curve: two-way repeated-measures ANOVA group: *F*_1,64_ = 53.84, *p* < 0.0001; trial: *F*_7,64_ = 8.80, *p* < 0.0001; interaction: *F*_7,64_ = 1.79, *p* = 0.10); inset: total number of entrances: *t*_8_ = 4.69, *p* = 0.002). **(B)** Ethosuximide treatment attenuated the learning impairment in the NVHL rats when compared to control rats (learning curve: two-way repeated-measures ANOVA group: *F*_1,64_ = 5.86, *p* = 0.02; trial: *F*_7,64_ = 9.79, *p* < 0.0001; interaction: *F*_7,64_ = 2.40, *p* = 0.03); inset: total number of entrances: *t*_8_ = 1.87, *p* = 0.10). **(C)** Olanzapine treatment did not improve performance of the NVHL rats on the two-frame task (learning curve: two-way repeated-measures ANOVA group: *F*_1,64_ = 42.87, *p* < 0.0001; trial: *F*_7,64_ = 9.66, *p* < 0.0001; interaction: *F*_7,64_ = 0.19, *p* = 0.99); inset: total number of entrances: *t*_8_ = 3.10, *p* = 0.02).

We then examined the effects on cognitive behavior. NVHL rats were slower to learn the place avoidance (Figure 8A), as we have previously reported (12). Ethosuximide attenuated the deficit because under ethosuximide NVHL rats performed at a level that was statistically indistinguishable from control rats, although there was a trend for them to be slower to learn in the initial trials (Figure 8B). The olanzapine treatment did not improve the cognitive performance of NVHL rats, which remained impaired compared to control rats (Figure 8C). In fact, olanzapine seemed to slightly impair avoidance of the control rats during the initial trials. This was confirmed by comparing the three groups of control rats from Figure 7 by two-way ANOVA (treatment: *F*_2,96_ = 9.70, *p* = 0.0001; trials: *F*_7,96_ = 33.86, *p* < 0.0001; interaction: *F*_14,96_ = 1.24, *p* = 0.26; *post hoc* tests: only olanzapine-treated control rats differed from ethosuximide-treated and untreated control rats).

**Figure 9:**
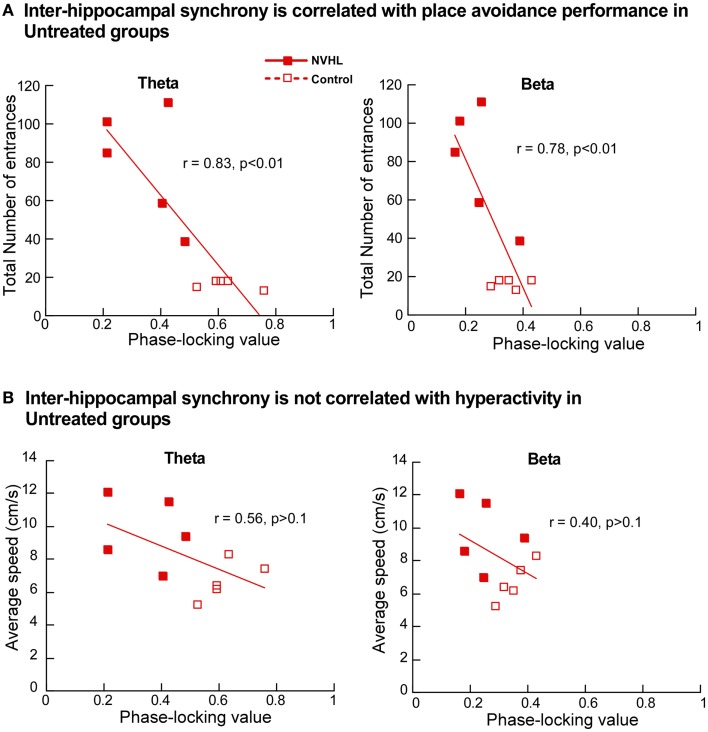
**Inter-hippocampal synchrony is correlated with place avoidance performance**. **(A)** In the untreated NVHL and control rats, the scatter plot distributions of theta phase-locking values (left) and beta phase-locking values (right) show significant correlation with the total number of errors in the two-frame avoidance task. **(B)** In the untreated NVHL and control rats, theta phase-locking values (left) and beta phase-locking values (right) were not correlated with hyperactivity, measured as running speed during the two-frame avoidance task.

Next we investigated the relationship between place avoidance performance and inter-hippocampal synchrony in the theta and beta bands. In the untreated NVHL and control rats, greater phase-locking at theta and beta frequencies was significantly correlated (*r*’s > 0.8; *p*’s < 0.01) with better place avoidance measured as lower total entrances (Figure 9A) but not with hyperactivity, measured as running speed (Figure 9B). The relationship between higher synchrony and lower entrances was apparent but not significant in the ethosuximide-treated animals (theta: *r* = 0.37; *p* > 0.1; beta: *r* = 0.14; *p* > 0.1). Because performance was good in most of these rats, there was limited variability in behavior for the synchrony measures to explain. The trend of a relationship between synchrony and cognition was reversed in the olanzapine-treated rats. Greater synchrony was associated with worse performance, but this trend was not significant (theta: *r* = 0.50; *p* > 0.1; beta: *r* = 0.35; *p* > 0.1).

**Figure 10:**
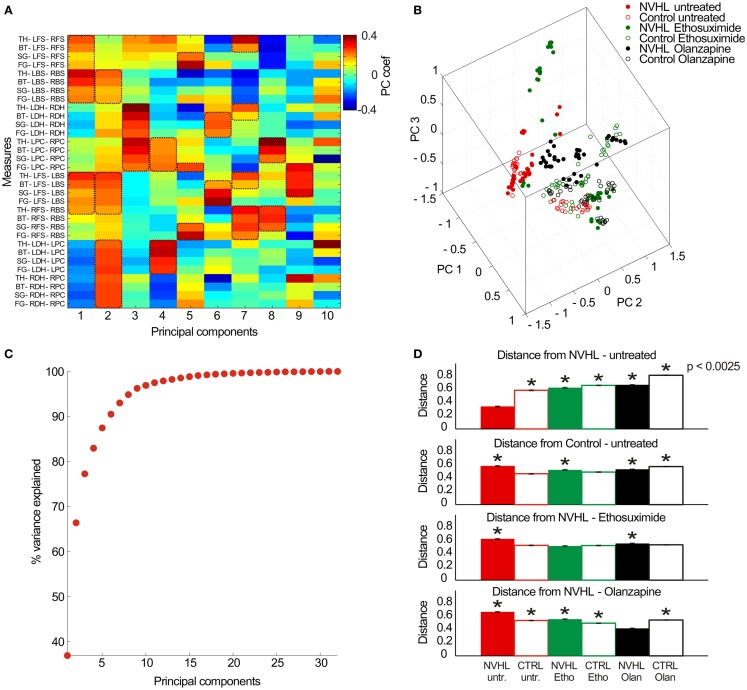
**Analysis of the multidimensional pattern of synchrony**. **(A)** Thirty-two PLV measures were extracted from the data (8 electrode pairs × 4 frequency bands) and treated as a set of 32-dimensional vectors. PCA analysis was performed on the resulting vector dataset. The first 10 principal components (PC) are shown here (*x*-axis) with the relative contribution of each measurement (*y*-axis) indicated by a color code. The PCs tend to group measures with similar properties. As an example, PC #1 corresponds mostly to frequency band-non-specific phase synchrony between the contralateral cortical electrode pairs as well as ipsilateral cortical electrode pairs, which are measures of global cortical phase synchrony. PC #3 corresponds mostly to frequency band-non-specific phase synchrony between the left and right-hippocampi as well as between the left and right prefrontal cortex, which is an estimate of region-specific inter-hemispheric synchrony. PC #6 corresponds to frequency-specific synchrony in the beta, slow gamma, and fast gamma bands between inter-hemispheric neocortical and hippocampal sites. The key measures of synchrony that contributed to each PC are indicated by the dashed boxes. **(B)** The multidimensional data can be visualized by plotting in the 3-D space of the first three PCs. **(C)** The cumulative probability of the dataset variance is explained by the PCs. The first six principal components explain ~90% of the dataset variance. **(D)** The average Euclidean distance between a pair of vectors estimates their distinctiveness. The distances between all pairs of vectors were computed within a treatment group and between all treatment groups and a specific treatment group to estimate similarity/distinctiveness of the treatment groups. The average distances are given to the untreated NVHL group (top row), to the untreated control group (second row), to the ethosuximide-treated NVHL group (third row), and to the olanzapine-treated NVHL group (bottom row).

Finally, in the effort to describe neural coordination during the place avoidance task, we examined the full set of 32 synchrony measures (8 electrode pairs × 4 frequency bands) as a pattern to investigate if there were group specific patterns of synchrony (Figure 10). The 32-dimensional space defined by the synchrony vectors (Figure 10A) was reduced to a 3-dimensional space to visualize the data using the first three principal components (Figure 10B). The data formed several clusters instead of filling the parameter space, which suggests neural activity may preferentially occupy preferred locales of this parameter state space. The untreated NVHL and control animals appeared to form two clusters. The ethosuximide and olanzapine-treated groups occupied distinct regions of the space away from the untreated groups. To estimate how distinct the groups might be, we computed the average distance amongst all pairs of vectors within a treatment group. The first six principal components accounted for over 90% of the variance in the data set (Figure 10C), so we computed the pair-wise distances as the 6-D Euclidean distance. The average distance from the untreated NVHL group was significantly greater for each group than it was for the vectors in the untreated NVHL group (Figure 10D). Similarly, the distances from the untreated control vectors were significantly greater for all other groups except the control group treated with ethosuximide, suggesting that ethosuximide did not significantly change control synchrony patterns. Because place avoidance behavior in the NVHL group was improved by ethosuximide, we estimated the distance between this group and the others. The distance to the untreated control group was no different than the distance within the NVHL-ethosuximide group, suggesting that the two groups were relatively similar. The complementary estimate of distance, this time to the olanzapine–NVHL group had a different answer. All groups had greater distances to the olanzapine–NVHL group than the distances amongst the group itself, consistent with the synchrony pattern caused by olanzapine being unique, rather than normalizing.

## Discussion

### Summary

The main finding of this study is that a drug that normalizes aberrant cognition-related neural synchrony in NVHL rats can also attenuate the cognitive control impairment in this schizophrenia-related neurodevelopmental model of mental dysfunction. This work demonstrates that the pattern of abnormal cognition-related neural synchrony is acutely normalized by ethosuximide, and furthermore shows that the synchrony alterations were associated with improved cognitive performance after ethosuximide but not olanzapine, despite both drugs being effective at reducing hyperactive locomotion in the NVHL model. Although olanzapine increased theta and beta synchrony between the two hippocampi, which is correlated with task performance (Figure 8), the drug also increased gamma synchrony beyond baseline levels, which may itself be associated with poor place avoidance performance in mutant mice (40, 41). Note that under ethosuximide, and also under olanzapine, the overall neural synchrony pattern in NVHL rats was distinct from the untreated control pattern, but the pattern after ethosuximide was closer to the control pattern than the pattern after olanzapine (Figure 10D) and perhaps this is one of the reasons that place avoidance performance was improved by ethosuximide but not olanzapine.

We have used the NVHL model to examine abnormal synchrony and cognitive impairment. Although NVHL rats are not a model of schizophrenia *per se*, these animals are an experimental model with neural synchrony and related cognitive abnormalities, which allows a prediction of the discoordination hypothesis to be tested. This unifying hypothesis was proposed to account for the schizophrenia syndrome independent of etiology (1, 2, 12, 27, 28). This view acknowledges that schizophrenia may turn out to be heterogeneous and that multiple factors contribute, including genetic alterations, infectious, toxic, and stressful events (42, 43). Whatever the etiology, the discoordination hypothesis asserts that disruption of the physiological synchronization of neural activities produces cognitive abnormalities in schizophrenia, despite otherwise seemingly normal expression of unitary physiological phenomena. The hypothesis is based specifically on the physiological coordination of neural activities, defined as the set of neural processes that control the timing of spike discharge across ensembles of neurons (1, 29, 44, 45). Our group has demonstrated this neural coordination during cognitive control in active place avoidance tasks (46, 47). The current findings support the discoordination hypothesis by demonstrating that correcting the neural synchrony abnormality in NVHL rats can be associated with cognitive improvement, which was the case with ethosuximide but not with olanzapine. Olanzapine did not normalize either neural synchrony or cognitive behavior (Figures 4, 5, and 8). In further support of the hypothesis, olanzapine caused abnormal neural synchrony in control rats, which seemed to worsen the place avoidance.

### Spike-wave activity

A secondary finding in this study is that spike-wave like activity was observed in the epidural cortical EEG of both NVHL and control rats during stillness in the home-cage and only rarely during active place avoidance behavior, consistent with prior observations (32, 34). This observation may have implications for how deficits in NVHL animals are interpreted when those deficits are characterized in tasks during which there is behavioral stillness, like pre-pulse inhibition, inhibitory avoidance, and food-motivated radial-arm mazes. Complex interactions of inhibitory and excitatory systems between the thalamus and cortical structures generate a variety of normal and abnormal rhythmic states including spindle waves and SWDs during absence seizures. Spindle waves are characterized by 1–4 s periods of 6–14 Hz oscillations during normal sleep (48). The characteristic frequency of the SWD is 3–4 Hz in humans (49) and is normally higher (4–12 Hz) in rodents (50, 51). Our analysis of cortical spike-wave activity shows that the features of the spike-wave activity in NVHL and control rats are different (Figure 2). The duration of the activity was greater in NVHL rats compared to control rats, further suggesting that this activity in the NVHL rats may be of a different neural origin than it is in control rats. In NVHL rats it may reflect a form of pathological network synchrony and even a sign of epileptiform propensity (52).

Indeed, during several hours of continuous monitoring, we have observed rare, but nonetheless *bona fide* epileptiform discharges and behavioral seizures in NVHL rats (52). The NHVL rats may have increased susceptibility to seizures due to dysregulation of the GABA system (53, 54). There is widespread reduction of GAD67 expression (54, 55), and decreased expression of PV interneurons in the prefrontal cortex and the hippocampus (54) in these rats. There is also increased firing in pyramidal neurons in the NVHL rats. The mPFC pyramidal neurons of NVHL rats respond to stimulation of the ventral tegmental area, the origin of dopamine projections to the PFC, with an increase in firing rates (56) instead of the normal decrease (57), indicating hyperexcitability. It has been shown that NVHL rats developed epileptiform SWDs at a twofold lower dose of pentylenetetrazol (PTZ, a GABA_A_ antagonist) than sham-operated rats, further evidence that the NVHL rats have increased susceptibility to absence-like seizures (54). Indeed, PD7 injections into ventral hippocampus with kainate, an excitotoxin with a different mechanism of action than ibotenate (58), is used as a model of temporal lobe epilepsy (59). Perturbations of the GABA system, together with the aberrant response reflecting the inability of dopamine to activate interneurons, may result in enhanced cortical excitability and abnormal spike-wave activity in NVHL rats.

### Ethosuximide treatment

We now discuss the effects of ethosuximide in the contexts of its pharmacological actions, typical use as well as the relevance of the findings with ethosuximide to understanding the cognitive impairments in the NVHL rat model and mental illness such as schizophrenia. To be clear, neither the findings nor the discussion that follows suggest that ethosuximide might be an effective treatment for schizophrenia. Rather, the procognitive and normalizing neural coordination effects of ethosuximide in the NVHL model provides new evidence that attenuating neural synchrony deficits can itself be procognitive.

Ethosuximide is used extensively to treat absence (petit mal) seizures (60–62). The mechanism of action is to block low-threshold Ca^2+^ currents in the T-type Ca^2+^ channels and enhance GABAergic tone (63, 64). We observed that ethosuximide reduced spike-wave activity in NVHL rats, indicating it is effective on abnormal synchrony in NVHL rats during home-cage behaviors (Figure 3). The drug also increased inter-hippocampal synchrony in NVHL rats so that neural coordination between the two hippocampi approached normal levels in the theta- and beta-frequency bands. Nonetheless, taken together, after ethosuximide, the overall pattern of neural synchrony in the NVHL rats was modified but the pattern of synchronies was not restored to the pattern of the untreated controls. Indeed, ethosuximide also changed the synchrony pattern in the controls animals too, but these changes were not associated with cognitive disability.

The effectiveness of ethosuximide may be contemplated in relation to the fact that the synchronization of oscillations reflects the temporally precise interaction of neural activities (65). Such coordinated interactions result from an appropriate balance between GABA-mediated inhibition and glutamate-mediated excitation, which may be unbalanced in adult NVHL rats (56). Alterations in one or both of these systems can result in abnormal network synchrony. Schizophrenia has been associated with dysregulation of cortical GABAergic neurotransmission (66) and abnormalities in NMDA-receptor mediated neurotransmission (67). There are significantly decreased levels of GABAergic neuronal markers like parvalbumin mRNA in the prefrontal cortex (68, 69) and in the dorsolateral prefrontal cortex of schizophrenia patients, decreased levels have been reported of glutamic acid decarboxylase (GAD67) mRNA, the GABA-synthesizing enzyme (70, 71). Indeed, NMDA-receptor hypofunction in schizophrenia (72, 73) is now thought to primarily affect GABA inhibition by reducing GABAergic tone. Thus dysregulation of excitatory and inhibitory neurotransmission contribute to abnormalities in the coordination of neural activities, which according to the hypothesis, is the basis for the impaired cognitive functions that are central to schizophrenia and a logical target for developing cognition promoting therapies. It was recently proposed that aberrant low frequency delta/theta oscillations emerge as a consequence of NMDA-receptor hypofunction (3, 74). The reduced excitation would deinactivate T-type Ca^2+^ channels and exaggerate bursting, especially in thalamic neurons, which may be the origin of the low frequency thalamocortical oscillations that could disrupt cortical function in schizophrenia (75). In this context, given ethosuximide’s mechanism of action, it is perhaps not surprising that the drug both normalized neural synchrony and improved cognition in NVHL rats. Ethosuximide reduces excitability primarily by blocking activity-dependent low-threshold Ca^2+^ currents as well as by acting as a partial agonist at the picrotoxin GABA-blocking receptor (64).

In the absence of the forgoing discussion, it may seem surprising to some readers that ethosuximide can improve inter-hippocampal synchrony and place avoidance behavior. Some readers may have assumed that because ethosuximide is antiepileptic it must decrease neural synchrony because epileptiform activity is thought of as excessive synchrony. According to these opinions, ethosuximide would decrease synchrony, not increase it, and thus worsen place avoidance behavior in control as well as NVHL rats. As we have pointed out in the Section “Introduction,” epileptiform activity is not simply excessive synchrony, and in the NVHL rat, we have measured the evidence of widespread decreases of synchrony between brain areas, resembling functional disconnection (52). Embedded in this loosening of neural coordination, perhaps by excitation–inhibition uncoupling, we also observed that some brain areas can exhibit relatively increased synchrony. Thus, there are alternative conceptualizations of the ethosuximide effects but they are not supported by the data on the subject in general or on NVHL rats in particular.

Ethosuximide treatment also reduced hyperactivity in NVHL rats, which in animal models, is widely studied as a behavioral analog of the positive symptoms of schizophrenia, although the reduction was not as effective as after olanzapine. Blocking T-type Ca^2+^ channels can produce antipsychotic effects in rats by attenuating the psychomotor effects of both the NMDA antagonist MK-801 and amphetamine, the dopaminergic psychostimulant (76). T-type Ca^2+^ channels are widely expressed in the brain, including areas such as the thalamus, the prefrontal cortex, the hippocampus, and the nucleus accumbens (77), which are regions that have been reported to function abnormally in schizophrenia (74, 78, 79). Interestingly, a number of clinically validated antipsychotics, including haloperidol, pimozide, flunarizine, and clozapine, are potent T-type channel antagonists (76, 80–82). A recent genome-wide study of single-nucleotide polymorphisms (SNP) found that variations in calcium channel genes are associated with schizophrenia as well as a range of major psychiatric disorders (83). These data implicate calcium channels in the pathophysiology of schizophrenia, and while calcium channel abnormality may help to account for why ethosuximide was effective on NVHL rats, it is also possible that ethosuximide was effective because of its fundamental effects on excitation–inhibition balance through the action on otherwise normal calcium channels. Nonetheless, the present work does not imply and we do not propose to use ethosuximide as a treatment for cognitive deficits in schizophrenia. Rather the present findings demonstrate that procognitive effects may be possible if treatments can normalize the coordination of neural activity between cognitive processing centers like the two hippocampi.

### Olanzapine treatment

Second-generation (atypical) antipsychotic medications, including olanzapine, are effective in treating psychosis, hallucinations, and delusions (84) but ineffective in treating the cognitive impairments that strongly debilitate schizophrenia patients (85). Olanzapine, a thienobenzodiazepine derivative, is classified as a multi-acting receptor-targeted antipsychotic, showing high affinity for dopaminergic, serotonergic, cholinergic, histaminergic, and muscarinic receptors (86, 87). As expected, we observed that olanzapine reduced hyperactivity in NVHL rats to control levels during the place avoidance task, indicating we used an effective antipsychotic dose, at least by traditional measures. The dose was however, ineffective at improving cognitive performance of NVHL rats in the two-frame task. This is of course consistent with the drug’s lack of effect on cognitive symptoms in patients.

Olanzapine treatment in NVHL and control rats showed a reversed effect on neural synchrony. Olanzapine increased inter-hippocampal synchrony in NVHL rats to the level of untreated controls in the theta and beta bands and in excess of the control levels in the gamma bands. In contrast, the drug decreased inter-hippocampal synchrony in control rats. The increased synchrony in olanzapine-treated NVHL rats was not associated with improved cognitive performance in the NVHL rats, which would be at odds with the discoordination hypothesis, except that after olanzapine, the gamma synchrony was excessive, indicating that olanzapine changed neural coordination, but as demonstrated with the multidimensional synchrony analysis (Figure 10), the synchrony changes did not normalize neural synchrony in NVHL rats. Conversely, but also in support of the discoordination hypothesis, the decreased synchrony in olanzapine-treated control rats was associated with a higher number of errors in the two-frame task when compared to untreated control rats, which is consistent with the observation that the drug changed the neural synchrony pattern in control animals to a pattern that is substantially distinct (Figures 10B,D) from the untreated control pattern. It is well documented that antipsychotic drugs cause EEG abnormalities associated with general slowing of background activity, an increase in paroxysmal theta or delta activity and the development of epileptiform discharges (88). In patients on olanzapine, there is an increased diffuse and intermittent slowing of the EEG (89, 90) and an increased high risk of EEG abnormalities such as theta and delta slowing, sharp waves or phase reversal, and/or spike-wave activities (88, 91). Healthy subjects on olanzapine showed an increased power in the theta band and a decrease in the beta band (92). In summary, olanzapine normalized inter-hippocampal synchrony in the theta and beta bands but it also caused abnormal synchrony changes in both the NVHL and control rats that were not associated with improved cognitive performance in the two-frame task.

## Conclusion

The findings reported here are consistent with the main assertion and prediction of the discoordination hypothesis that abnormal neural synchrony during cognitive effort is associated with impaired cognition and that restoring normal synchrony will promote cognition. As shown here, targeting the pathophysiology of abnormal neural coordination, regardless of the etiology, may be both a rational and effective program for developing a much-needed generation of procognitive treatments for mental illness in general and schizophrenia in particular.

## Author Contributions

Heekyung Lee collected and analyzed data, Dino Dvorak analyzed data, Heekyung Lee and André A. Fenton wrote the manuscript, and André A. Fenton planned and organized the research.

## Conflict of Interest Statement

The authors declare that the research was conducted in the absence of any commercial or financial relationships that could be construed as a potential conflict of interest.
